# Supplementary material for: Characterization of the Bacteriome of Culicoides reevesi from Chihuahua, Northern Mexico: Symbiotic and Pathogenic Associations
Source: Insects. 2026 Jan 1;17(1):52. doi: 10.3390/insects17010052 (PMC12841952; doi:10.3390/insects17010052)
Supplement: Supplementary file 1 [file insects-17-00052-s001.zip › Supplementary Material C reevesi bacteriome.pdf]

Supplementary Material: Taxonomic Resolution and Unclassified Fractions — *Culicoides reevesi* Bacteriome

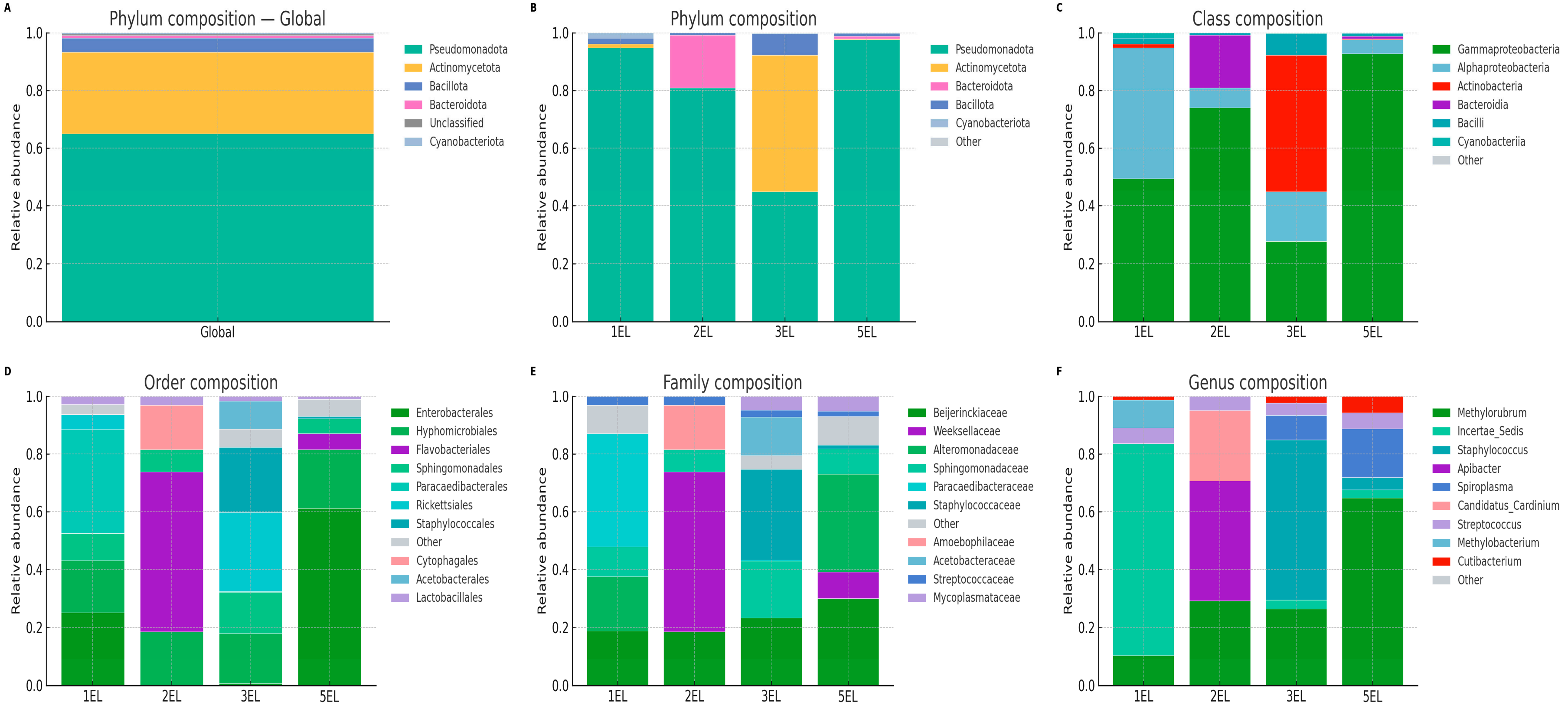

Figure S1. Rank-resolved bacterial composition of *Culicoides reevesi*. (A) Global relative abundance at the Phylum across all pools. (B–F) Pool-specific relative abundances were summarized across Phylum, Class, Order, Family, and Genus (1EL, 2EL, 3EL, 5EL). Stacked bars show relative abundances; low-abundance taxa were grouped as Others. Species-level assignments were not included because the SILVA 138–99 classifier for 515F/806R is validated up to Genus

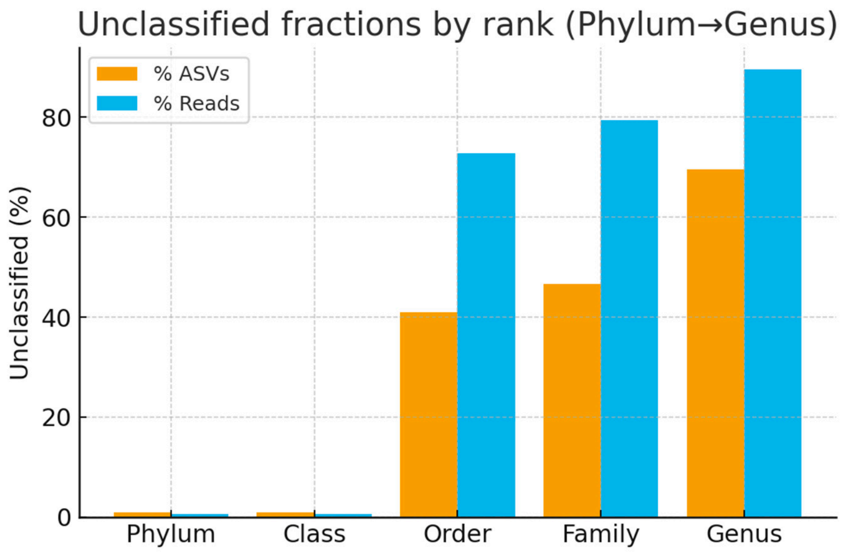

Figure S2. Unclassified fractions by rank (Phylum→Genus). Percentage of *Unclassified* assignments summarized across pools, reported by ASVs (orange) and by reads (blue). Bars show the fraction at each taxonomic rank from Phylum to Genus.

Supplementary Material – Diversity Analysis of the *Culicoides reevesi* bacteriome

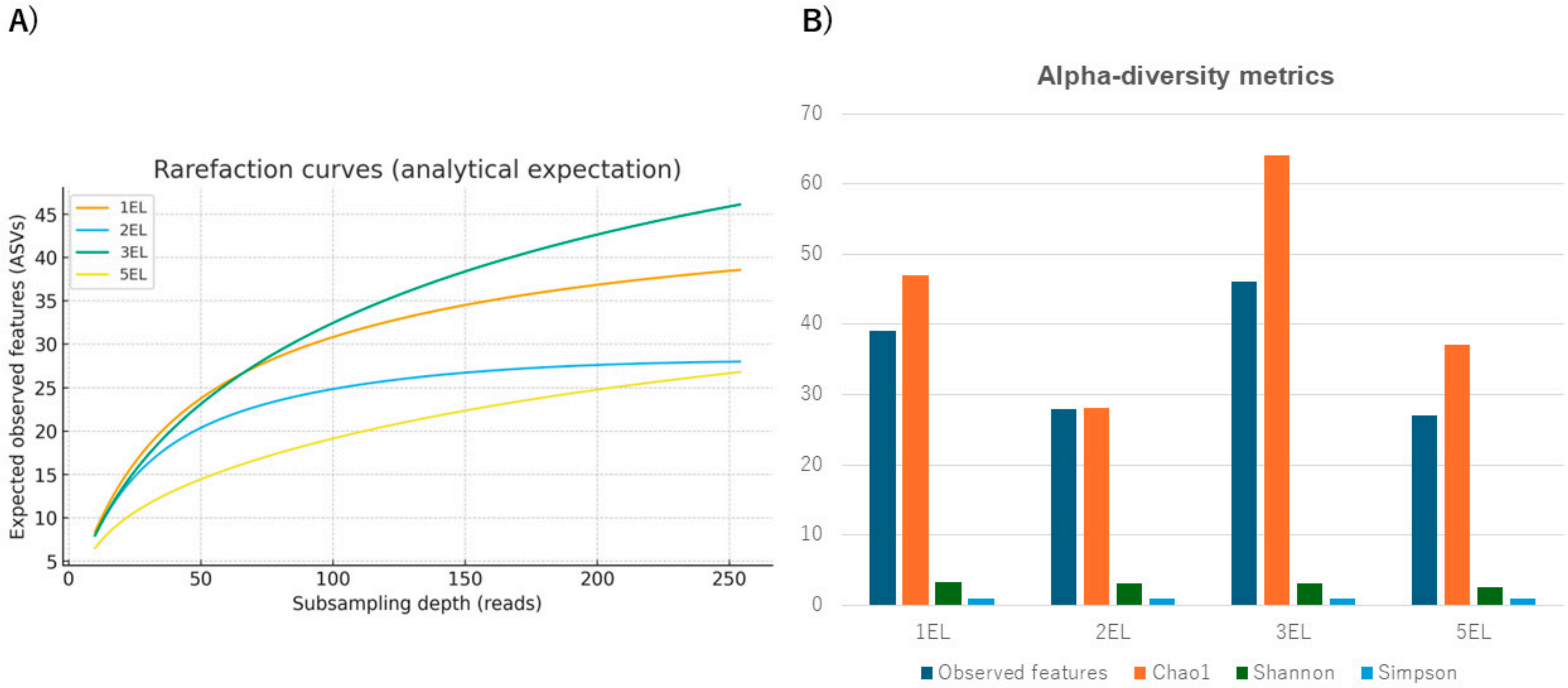

Figure S3. Rarefaction and alpha diversity. Rarefaction curves of expected Observed ASVs up to a subsampling depth of 254 reads. (B) Alpha-diversity at depth 254 (Observed, Chao1, Shannon, Simpson [1–D]) for pools 1EL, 2EL, 3EL, and 5EL. Values were calculated from the rarefied feature table (depth = 254).

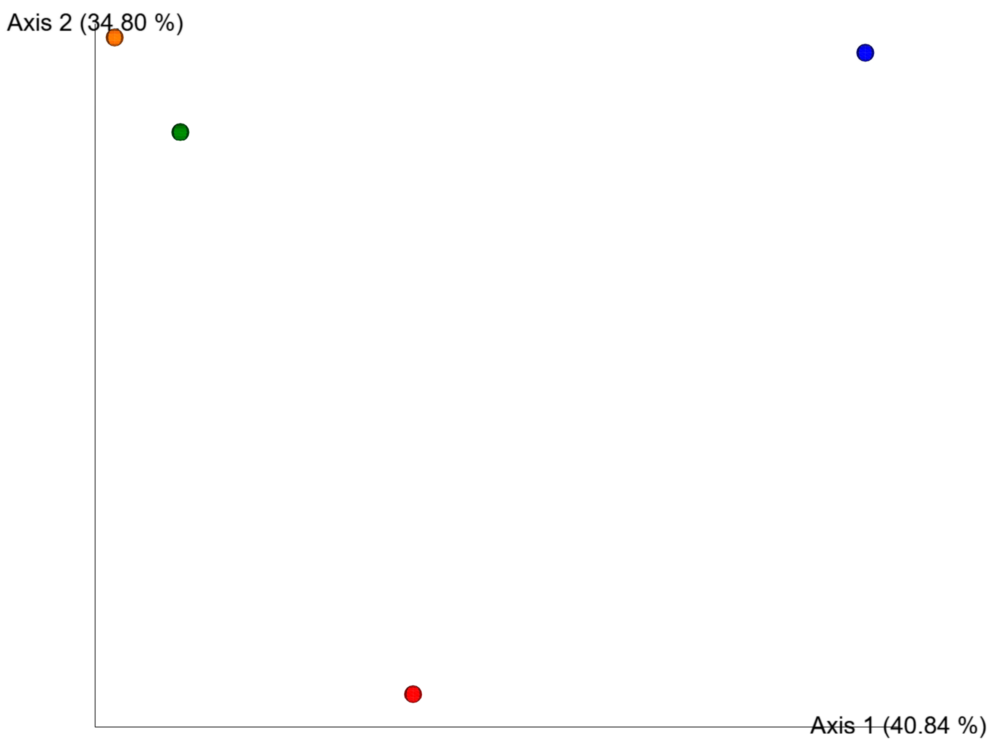

Figure S4. Principal coordinates analysis (PCoA) based on Bray–Curtis dissimilarity. Axis labels indicate the proportion of variance explained.
